# Extra-Nuclear Signaling of Progesterone Receptor to Breast Cancer Cell Movement and Invasion through the Actin Cytoskeleton

**DOI:** 10.1371/journal.pone.0002790

**Published:** 2008-07-30

**Authors:** Xiao-Dong Fu, Maria S. Giretti, Chiara Baldacci, Silvia Garibaldi, Marina Flamini, Angel Matias Sanchez, Angiolo Gadducci, Andrea R. Genazzani, Tommaso Simoncini

**Affiliations:** 1 Molecular and Cellular Gynecological Endocrinology Laboratory (MCGEL), Department of Reproductive Medicine and Child Development, University of Pisa, Pisa, Italy; 2 Department of Physiology, Zhongshan School of Medicine, Sun Yat-sen University, Guangzhou, China; Ordway Research Institute, United States of America

## Abstract

Progesterone plays a role in breast cancer development and progression but the effects on breast cancer cell movement or invasion have not been fully explored. In this study, we investigate the actions of natural progesterone and of the synthetic progestin medroxyprogesterone acetate (MPA) on actin cytoskeleton remodeling and on breast cancer cell movement and invasion. In particular, we characterize the nongenomic signaling cascades implicated in these actions. T47-D breast cancer cells display enhanced horizontal migration and invasion of three-dimensional matrices in the presence of both progestins. Exposure to the hormones triggers a rapid remodeling of the actin cytoskeleton and the formation of membrane ruffles required for cell movement, which are dependent on the rapid phosphorylation of the actin-regulatory protein moesin. The extra-cellular small GTPase RhoA/Rho-associated kinase (ROCK-2) cascade plays central role in progesterone- and MPA-induced moesin activation, cell migration and invasion. In the presence of progesterone, progesterone receptor A (PRA) interacts with the G protein Gα_13_, while MPA drives PR to interact with tyrosine kinase c-Src and to activate phosphatidylinositol-3 kinase, leading to the activation of RhoA/ROCK-2. In conclusion, our findings manifest that progesterone and MPA promote breast cancer cell movement via rapid actin cytoskeleton remodeling, which are mediated by moesin activation. These events are triggered by RhoA/ROCK-2 cascade through partially differing pathways by the two compounds. These results provide original mechanistic explanations for the effects of progestins on breast cancer progression and highlight potential targets to treat endocrine-sensitive breast cancers.

## Introduction

Estrogen is regarded as a carcinogenic factor in the breast [1] and have recently found that estrogen may also alter breast cancer progression by promoting tumour cells motility [2]. However, the role of progesterone receptor (PR) signaling in the development and progression of breast cancer is poorly characterized notwithstanding its relevance in the clinical setting [1]. To this extent, the Multiethnic Cohort and Women's Health Initiative trials show an increased incidence of breast cancer in postmenopausal women receiving combined hormone therapy with estrogens and progestogens as compared to the women receiving estrogens alone, suggesting that progestins may play a deleterious role on breast cancer [3]–[5].

Local breast cancer spread and its later diffusion to the lymph nodes or to distant sites are the main cause of morbidity and death [6]. The generation of cancer cell movement in the surrounding environment is the first step in these processes and involves a complex set of cellular actions. A critical step is represented by the remodeling of the actin cytoskeleton toward the cell membrane, which allows the formation of bridges between the backbone of the cell and the extracellular matrix mediated by anchorage proteins. The ensuing contractions of the cytoskeleton generate cell movement [7]. Actin remodeling (particularly the loss of stress fibres) is also involved in cancer transformation and metastasis [8].

Moesin, a member of the ezrin/radixin/moesin (ERM) family, is an actin-binding protein that plays an important role in cell motility by linking the actin cytoskeleton to a variety of membrane-anchoring proteins [9], [10]. In quiescent conditions moesin exists in an auto-inhibited conformation and phosphorylation of Thr^558^ within the C-terminal actin binding domain by the Rho-associated kinase (ROCK), results in a conformational change and in the association with the scaffold protein, ezrin/radixin/moesin-binding protein 50 (EBP50) on moesin's NH_2_-terminal end and with F-actin on moesin's COOH-terminal end to mediate the linkage of microfilaments to membranes in cell surface microvilli [11].

We recently showed that estrogen controls actin remodeling in endothelial cells via the activation of moesin [12]. This ensues through a rapid, extra-nuclear signaling cascade originated by the interaction of ERα with the G protein Gα_13_. This process leads to the recruitment of RhoA and of the Rho associated kinase, ROCK-2 and to moesin activation. This pathway leads to the formation of membrane ruffles and pseudopodia which interact with the extracellular matrix and with nearby cells, thus promoting cell migration [12]. Moreover, the activation of this pathway mediates estrogen-induced migration and invasion of breast cancer cells [2].

The pharmacological properties of progestins are not equal [13], [14]. These pharmacological discrepancies may account for the diverse impact of progestins on breast cancer development and progression. For instance, the E3N-EPIC cohort study show that continuous-combined HRT with different progestins is associated with the different relative risk and subtype of breast cancer in postmenopausal women [15], [16]. Hence it would be clinically important to be able to differentiate the effects on breast cells of the different progestins used for HRT.

The sex steroid progesterone and the various synthetic progestins act in human cells through progesterone receptor (PR) A and PRB [17]. Beyond being transcription factors actively involved in the regulation of gene expression, PRs also act via rapid, extra-nuclear, signaling cascades, such as via the phosphatidylinositol 3-kinase (PI3K)/Akt or the c-Src/extracellular signal-regulated kinases 1/2 (ERK1/2) pathways, playing an important role in breast cancer development [18], [19]. However, little is known on the functional relevance of PR signaling for breast cancer progression.

In this manuscript we investigate the regulatory actions of PR on breast cancer cell migration and invasion and we characterize the extra-nuclear signaling events recruited by PR.

## Results

### Progesterone and MPA drive breast cancer cell migration and invasion

**Figure 1:**
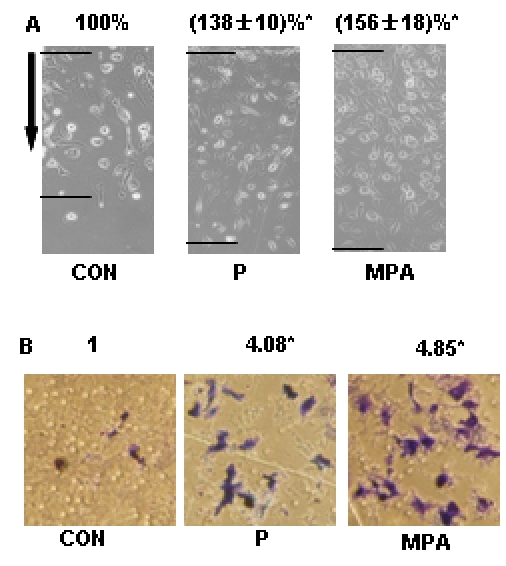
PR activation increases T47-D cell migration and invasion. (A) Cells were treated with progesterone or MPA (both 100 nM) for 48 h and cell migration was assayed. T47-D cells were scraped out of the cell culture dish and the extent of migration of the remaining cells was assayed in the presence of Ara-C (see text). Cell migration distances were measured and values are presented as % of control. * = P<0.01 vs. control. The experiments were performed in triplicates and data representing the migration distance of cells from the starting line are expressed as mean±SD. The arrows indicate the direction of migration. The upper black lines indicate the starting line and the lower black lines indicate the mean migration distance. (B) Cells were treated with progesterone or MPA (both 100 nM) for 48 h. Cell invasion was assayed using invasion chambers. Invading cells were counted in three different central fields of triplicate membranes. The experiments were performed in triplicates. Invasion indexes and representative images are shown. * = P<0.01 vs. control.

First we observed the actions of progesterone and MPA on T47-D breast cancer cell migration and invasion. Before treatment, T47-D breast cancer cells were pretreated with cytosine β-D-arabinofuranoside hydrochloride (Ara-C - 10 µM) to prevent cell division. Activation of progesterone receptor (PR) with either natural progesterone (P, 100 nM) or the synthetic progestin medroxyprogesterone acetate (MPA, 100 nM) resulted in enhanced migration vs. vehicle-treated cells (Fig. 1A). In the inserts with GFR Matrigel, recruitment of PR with P or MPA promoted invasion of the matrix by cancer cells (Fig. 1B). The number of cells that invaded the matrix in the presence of MPA was higher than with P (Fig. 1B).

### Rapid activation of PR is linked to breast cancer cell cytoskeletal and cell membrane rearrangement

**Figure 2:**
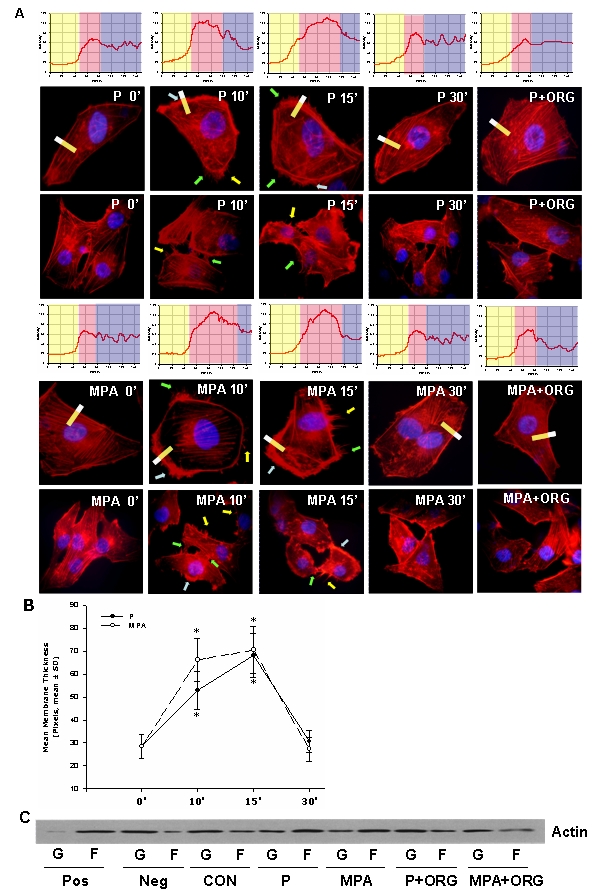
PR activation induces a rapid rearrangement of the actin cytoskeleton in T47-D cells. (A) T47-D cells were treated with P or MPA (both 100 nM) for 10, 15 or 30 minutes, in the presence or absence of the pure PR antagonist ORG 31710 (1 µM). Immunofluorescent analysis of Texas Red-phalloidin (in red) reveals the spatial modifications of actin fibres through the time-course and the formation of specialized cell membrane structures. Green, yellow and light blue arrows indicate lamellipodia, pseudopodia and ruffles, respectively. Nuclei are counterstained in blue. Rectangles indicate the area sampled in the corresponding upper graph. In the graph, the longitudinal axis displays the gray level and the horizontal axis shows the pixels. Light yellow, light red and light blue areas indicate the parts of the graph indicating the extracellular, plasma membrane and cytoplasmic areas. (B) Analytic results obtained by using Leica QWin image analysis and processing software showing the mean thickness of the cell membrane after treatment with P or MPA (both 100 nM). The results are derived from the sampling of five areas of the cell membrane of thirty different random cells. The areas of minimum and maximum cell membrane thickness were always included. The results are the mean±SD of the measurements. (C) shows the amount of filamentous actin (F-actin, F) versus free globular-actin (G-actin, G) content in T47-D cells after treatment with P or MPA (both 100 nM) for 15 min, in the presence or absence of PR antagonist ORG 31710 (1 µM). Positive (Pos) and negative (Neg) controls were set by adding F-actin enhancing solution (phalloidin, 1 µM) or F-actin depolymerization solution (10 µM cytochalasin-D) to the lysates, respectively. All the experiments were repeated three times with consistent results, and a representative result is shown. * = P<0.05 vs control.

Actin fibers in ER^+^/PR^+^ T47-D breast cancer at baseline were arranged longitudinally in the cytoplasm and the cell membrane was regular. Activation of PR with P (100 nM) or MPA (100 nM) resulted in a rapid shift of the actin fibers toward the edge of the membrane. This was associated with a significant increase of the thickness of the cell membrane and of its fluorescence intensity, quantified by analyzing the pixel intensity in a box including the cell membrane as well as the adjacent intra- and extra-cellular space (Fig. 2A, 2B and Table 1). In parallel, cell membrane ruffles and pseudopodia were formed at sites enriched in actin (Fig. 2A). These effects were maximal between 10 and 15 minutes and began to revert after 30 minutes (Fig. 2A, 2B and Table 1). These processes were prevented by the pure PR antagonist ORG 31710 (Fig. 2A, 2B and Table 1).

**Table 1:** The table displays the mean thickness of the cell membrane, the mean actin intensity of the membrane and the cytoplasm, as well as the ratio of the intensities of membrane/cytoplasm in T47-D cells treated with progesterone and MPA (both 100 nM) for different times, in the presence or absence of PR antagonist ORG 31710 (1 µM).

		Mean membrane thickness (pixel±SD)	Mean membrane intensity (mean gray level±SD)	Mean cytosol intensity (mean gray level±SD)	Membrane/cytosol intensity ratio
0′		28.4±5.2	60.3±7.8	58.6±6.6	1.04
10′	P	52.8±8.3*	98.4±10.2*	60.3±7.2	1.63*
	MPA	66.2±9.4*	97.5±8.6*	64.5±8.4	1.61*
15′	P	68.3±9.6*	104.6±11.5*	62.4±8.3	1.74*
	MPA	70.5±10.3 *	106.8±10.6*	56.4±9.1	1.86*
30′	P	30.6±4.7	70.4±8.1	61.6 ±7.2	1.13
	MPA	27.3±5.2	60.5±9.4	58.3±4.8	1.03
15′+ORG	P	27.5±5.6	58.2±6.9	60.4±7.5	0.98
	MPA	30.6±4.4	64.3 ±7.2	56.1±5.5	1.27

Analytic results were obtained by using Leica QWin image analysis and processing software.

When the globular/fibrillar (G/F) actin ratio was assayed in T47-D cells, similar changes were observed. At baseline, actin predominantly existed as monomers (G-actin), while after recruitment of PR with P or MPA for 15 min, a rapid shift toward F-actin was found that was prevented by ORG 31710 (Figure 2C), indicating that PR activation is linked to rapid actin polymerization. The amount of total actin (G-actin+F-actin) was comparable in all conditions (Figure 2C).

### Recruitment of PR leads to activation of the actin-regulatory protein, moesin

**Figure 3:**
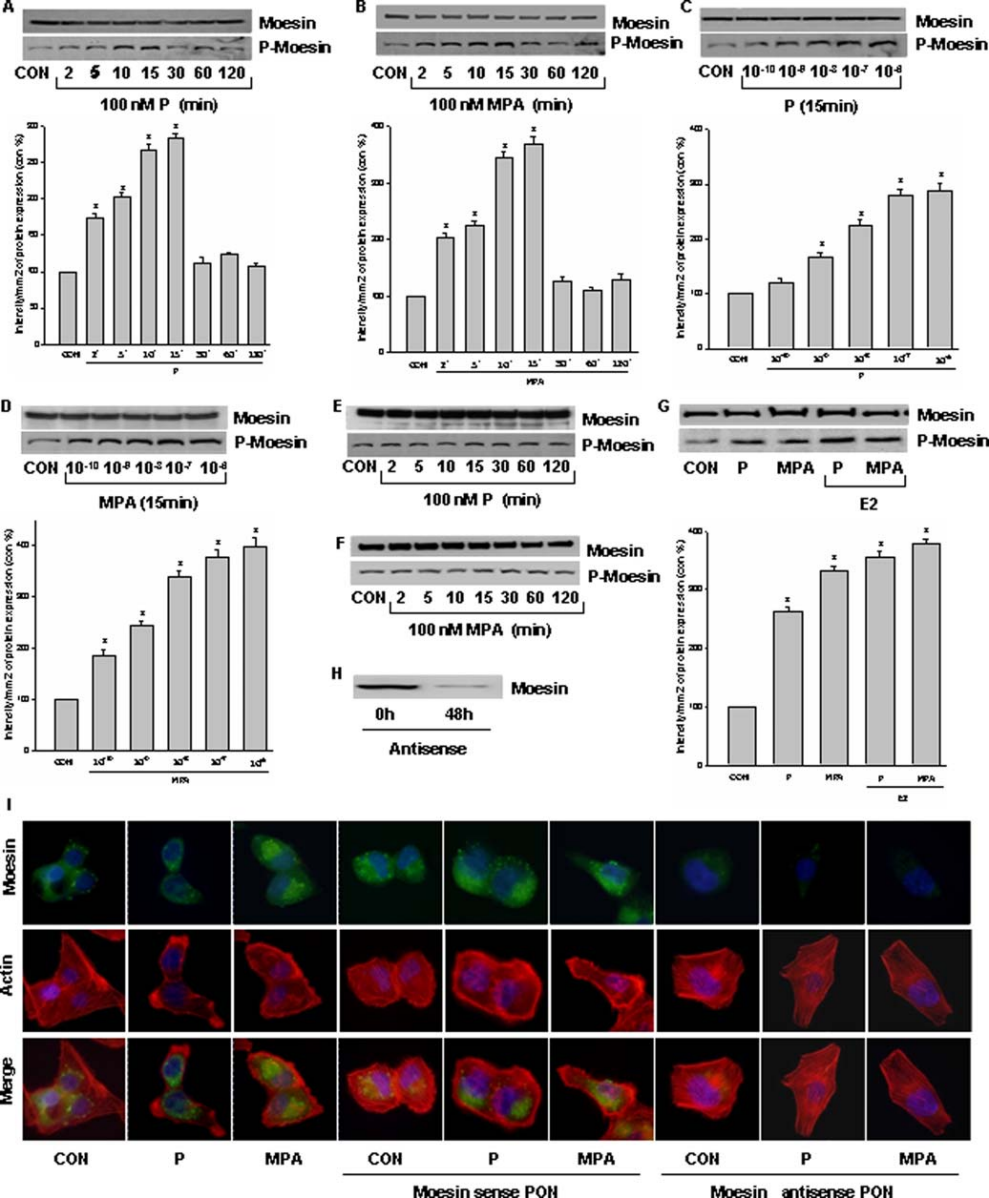
PR activation turns into moesin activation. (A), (B) (C) and (D) show the time- and dose-dependent moesin activation in T47-D breast cancer cells after recruitment of PR with P or MPA. Total cell amount of wild-type (Moesin) or Thr^558^-phosphorylated moesin (P-Moesin) are shown with western blot. * = P<0.05 vs control. (E) and (F) show that in MDA-MB-231 cells (that do not express PR), progesterone and MPA have no effect on moesin activation. (G) shows that moesin activation induced by progestins alone or in combination with 17β-estradiol (E2). * = P<0.05 vs control. (H) Moesin expression detected by western blot in T47-D cells transfected with moesin antisense PON for 48 h. (I) Actin remodeling after PR activation with P or MPA for 15 min in T47-D cells after transfection with moesin antisense phosphorotioate oligonucleotides (PON) (antisense - 2 µM) or sense PON (sense - 2 µM) for 48 h. Cells were stained with an Ab vs. moesin (FITC; green staining) as well as with Texas Red-phalloidin (in red). Nuclei are counterstained in blue. All the experiments were repeated three times with consistent results, and a representative result is shown.

Moesin rapidly increased in T47-D cells exposed to P (100 nM) or MPA (100 nM) between 2 (mean increases of moesin phosphorylation: P 73%, MPA 102%) and 15 minutes (mean increases of moesin phosphorylation: P 183%, MPA 268%) and then declined after 30 minutes, time-consistently with the kinetics of actin rearrangement (Fig. 3A–B). Moesin activation was related to concentration of the PR agonists (Fig. 3C–D). Supporting the requirement of PR, the same PR agonists did not alter moesin phosphorylation in MDA-MB-231 breast cancer cells, that do not express PR (Fig. 3E–F). In addition, moesin phosphorylation was slightly increased by the addition of E2 (1 nM) to each progestin compared to the progestins alone, although this was not statistically significant (Fig. 3G).

To establish the requirement of moesin for the PR-induced actin reorganization in T47-D cells we silenced moesin expression by transfecting specific antisense phosphorotioate oligonucleotides (PONs). After exposure to antisense moesin PONs for 48 h, moesin protein expression (Fig. 3H) and cell immunostaining (Fig. 3I) in T47-D cells were greatly reduced. Moesin-silenced T47-D cells did not respond with actin or cell membrane remodeling when PR was recruited by either P or MPA (Fig. 3I). As control, non-transfected T47-D cells or cells receiving sense (inactive) moesin PONs displayed a visible cytoskeletal and cell membrane reorganization in response to PR recruitment (Fig. 3I).

### Characterization if the initiation of PR signaling to moesin

**Figure 4:**
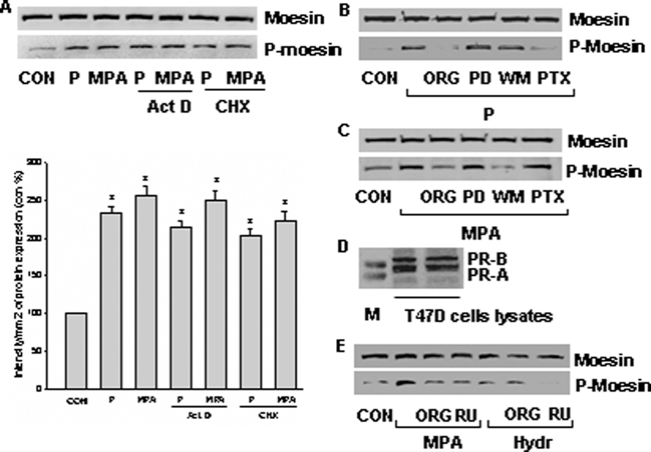
Extra-nuclear signaling of PR to moesin in T47-D cells. (A) T47-D cells were treated with progesterone or MPA (both 100 nM) for 15 min, in the presence or absence of Act D (10 µM) or CHX (200 µM). Moesin and phosphorylated moesin are shown. * = P<0.05 vs control. (B) and (C) Cells were exposed to 100 nM progesterone or MPA for 15 min, in the presence or absence of the pure PR antagonist ORG 31710 (ORG - 1 µM), of the MEK inhibitor PD98059 (PD - 5 µM), of the PI3K inhibitor wortmannin (WM - 30 nM) or of the G protein inhibitor, PTX (100 ng/mL). Cell contents of wild-type or phosphorylated moesin are shown. (D) The expression of PRA and PRB in T47-D cell lysates is shown (M - marker proteins). (E) Cells were exposed to 100 nM MPA or 50 nM hydrocortisone (Hydr) for 15 min, in the presence or absence of ORG 31710 (ORG - 1 µM) or of the combined GR/PR antagonist, RU486 (RU - 1 µM), moesin and phosphorylated moesin are assayed with western analysis. The experiments were performed in triplicates and representative images are shown.

The rapid time lapse of moesin activation and deactivation suggests that PR signals to this protein via “nongenomic” or “extra-nuclear” cascades [20]. Indeed, activation of PR with either P or MPA still resulted in moesin activation even if RNA or protein synthesis was blocked in T47-D cells with actinomycin D (Act D - 10 µM) or cycloheximide (CHX - 200 µM) (Fig. 4A).

Blockade of PR with ORG 31710 completely abolished both P- and MPA-dependent moesin activation, confirming that PR is the steroid receptor used by these agonists to signal to moesin (Fig. 4B–C). Interference with the ERK1/2 cascade with PD98059 did not alter the activation of moesin (Fig. 4B–C). Interestingly, inhibition of G proteins with pertussis toxin (PTX) prevented the activation of moesin by P but not by MPA, while inhibition of phosphatidylinositol-3OH kinase (PI3K) with wortmannin exclusively blocked the action of MPA but not that of P (Fig. 4B–C). These findings indicate that PR signals to moesin via a G protein-dependent pathway when bound by P, and via a PI3K-dependent pathway in the presence of MPA.

PR ligands exert their actions through the two PR isoforms, PRA and PRB, which are both expressed by the T47-D cells used in this study (Fig. 4D). However, in contrast to P, MPA also binds the glucocorticoid receptor (GR), that mediates some of its actions [21], [22]. This different receptor binding pattern explains some of the biological differences of the two compounds [21], [22]. However, phosphorylation of moesin in the presence of MPA was equally prevented by the pure PR antagonist ORG 31710 as well as by the mixed PR/GR antagonist RU486, suggesting that GR does not play a role in MPA signaling to moesin (Fig. 4E). In agreement, moesin was not phosphorylated in the presence of hydrocortisone (50 nM) (Fig. 4E).

**Figure 5:**
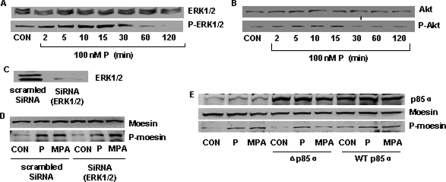
Extra-nuclear signaling of PR to moesin in T47-D cells: ERK1/2 and PI3K. (A) shows wild-type (ERK1/ERK2) or phosphorylated ERK 1/2 (P-ERK1/ERK2) during exposure to progesterone (100 nM). (B) Shows wild-type (Akt) and phosphorylated Akt (P-Akt) in the presence of 100 nM P. (C) T47-D cells were transfected with scrambled siRNA or ERK1/2 targeted siRNAs for 48 h. After that level of ERK1/2 protein expression was detected by western blot as indicated. (D) Cells were exposed to 100 nM P or MPA for 15 min after transfection with 100 nM targeted siRNA for ERK1/2 or scrambled siRNA for 48 h. Cell contents of wild-type or phosphorylated moesin are shown. (E) Cells were exposed to 100 nM progesterone or MPA for 15 min after transfection with constitutively active p85α (WT p85α) or dominant-negative p85α (Δp85α) for 48 h. Cell contents of p85α, wild-type or phosphorylated moesin are shown. The experiments were performed in triplicates and representative images are shown.

PR activation of ERK1/2 and PI3K in breast cancer cells is associated with cell proliferation and inhibition of apoptosis [18], [23]. Consistent with these reports, exposure of T47-D cells to P resulted in a time-dependent activation of ERK1/2 and of the PI3K effector, Akt (Fig. 5A–B).

However, recruitment of PR with either P or MPA in T47-D cells after silencing of ERK 1/2 with siRNAs (Fig. 5C) still resulted in activation of moesin (Fig. 5D), confirming that signaling of PR to the ERK1/2 mitogen-activated protein kinase (MAPK) cascade is not implicated in moesin activation.

Transfection of T47-D cells with a dominant negative form of the regulatory subunit of PI3K, p85α (Δp85α), resulted in the impairment of the PR-dependent moesin activation induced by MPA, but had no inhibitory effect on P (Fig. 5E), implying that PI3K only plays a role in PR signaling to moesin induced by MPA. As control, the transfection of a wild-type p85α construct (WT p85) did not alter moesin activation induced by P nor MPA (Fig. 5E).

**Figure 6:**
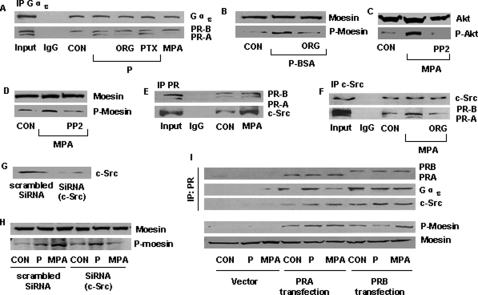
Extra-nuclear signaling of PR to moesin in T47-D cells: Gα_13_ and c-Src. (A) T47-D cells were treated for 15 minutes with P or MPA (both 100 nM), in the presence or absence of ORG 31710 (ORG - 1 µM) or of PTX (100 ng/mL). Protein extracts were immunoprecipitated with an Ab *vs*. Gα_13_ and the IPs were assayed for co-immunoprecipitation of PRs. The cell extract (30 µg) was used as input and normal rabbit IgG was used as the control antibody. (B) T47-D cells were exposed to 100 nM P-BSA (membrane-impermeable) for 15 min, in the presence or absence of ORG 31710 (ORG - 1 µM). Moesin and phosphorylated moesin are shown. (C) and (D) T47-D cells were treated with 100 nM MPA for 15 min, with or without the Src kinase inhibitor, PP2 (10 µM) and wild-type or active Akt or moesin are shown. (E) and (F) T47-D cells were treated for 15 minutes with 100 nM MPA, in the presence or absence of ORG 31710 (ORG - 1 µM). Protein extracts were immunoprecipitated with an Ab vs. PR (E) or c-Src (F) and the IPs were assayed for co-immunoprecipitation of PR or c-Src as indicated. Cell extract (30 µg) was used as input. Normal rabbit IgG and normal mouse IgG were used as the control antibodies in (E) and (F), respectively. (G) T47-D cells were transfected with scrambled siRNA or c-Src targeted siRNAs for 48 h. c-Src protein expression was detected by western blot as indicated. (H) T47-D cells were exposed to 100 nM P or MPA for 15 min after transfection with c-Src siRNA or non-specific control siRNAs for 48 h. Total moesin or P-moesin cell amounts are shown. (I) PR-negative MDA-MB-231 cells were transiently transfected with empty pcDNA3.1+ plasmid (vector) or plasmids encoding full length of human PRA or PRB for 48 h, then cells were exposed to 100 nM P or MPA for 15 min. Protein extracts were immunoprecipitated with an Ab vs. PR, and the IPs were assayed for co-immunoprecipitation of Gα_13_ or c-Src as indicated. Total moesin and phosphorylated moesin were also analyzed using western blot. The experiments were performed in triplicates and representative images are shown.

The G protein Gα_13_ is an established controller of the cytoskeleton and of cell movement [24]. Co-immunoprecipitation studies showed that, in the presence of progesterone, PRA started to interact with Gα_13_. The PRA/Gα_13_ interaction was ligand-dependent, being prevented by ORG 31710, but not by PTX (Fig. 6A). In addition, the PRA/Gα_13_ interaction was ligand-specific, as it was not triggered by MPA (Fig. 6A). Differently from PRA, a basal interaction between PRB and Gα_13_ was found, which was not altered by the addition of either P or MPA (Fig. 6A).

As G proteins reside on the cell-membrane and sub-sets of PRs have also been identified at this level [25], [26], we used the membrane-impermeable bovine serum albumin-progesterone conjugate (P-BSA - 100 nM) to explore if binding of PR at the cell membrane may be involved in moesin activation. Indeed, exposure of T47-D cells to P-BSA resulted in rapid activation of moesin (Fig. 6B).

PR interacts with the tyrosine kinase c-Src [27], and this process is involved in the activation of PI3K [28]. We thus explored the role of c-Src for the PR-dependent activation of moesin induced by MPA. Administration of MPA to T47-D cells lead to activation of the PI3K target Akt and of moesin, both of which were prevented by the Src kinase inhibitor, PP2 (Fig. 6C–D). Activation of Akt and moesin were associated with a ligand-induced interaction of both PRA and PRB with c-Src (Fig. 6E–F).

Silencing of c-Src with specific siRNAs (Fig. 6G) impaired the activation of moesin by MPA (Fig 6H). In contrast, P was still able to trigger moesin phosphorylation in c-Src-silenced cells (Fig. 6H), reinforcing the hypothesis that PR signaling to moesin is ligand-specific, and that the interaction with c-Src and the subsequent recruitment of the PI3K/Akt pathway are absolutely required when PR is engaged by MPA, but not in the presence of P.

To further differentiate the role of the two PR isoforms, we transfected full-length human PRA or PRB in ER^−^/PR^−^ MDA-MB-231 breast cancer cells and studied the interaction with Gα_13_ or c-Src and the activation of moesin. In MDA-MB-231 cells transfected with the vector plasmid pcDNA3.1+, baseline or ligand-associated interaction of PR with Gα_13_ or c-Src was negligible and no moesin activation was observed (Fig. 6I). In MDA-MB-231 cells transfected with PRA, P (but not MPA) enhanced the interaction of PRA with Gα_13_ (Fig. 6I). In the same cells enhanced interaction of PRA with c-Src was found in the presence of both P and MPA (Fig. 6I). In this experimental condition, exposure to P as well as to MPA was associated with increased moesin phosphorylation (Fig. 6I).

When MDA-MB-231 cells were transfected with PRB, a visible co-interaction of this receptor with Gα_13_ was seen, which was not altered by the presence of the ligands (Fig. 6I). Interaction of PRB with c-Src was instead dependent on the presence of either P or MPA (Fig. 6I). However, in these cells, only exposure to MPA resulted in activation of moesin.

Overall, these results indicate that signaling to moesin in T47-D cells is initiated through a PRA/Gα_13_ interaction in the presence of progesterone, or alternatively through a PRA/B-dependent recruitment of c-Src when MPA is present.

### Later intracellular events linking activation of PR to moesin: role of RhoA

**Figure 7:**
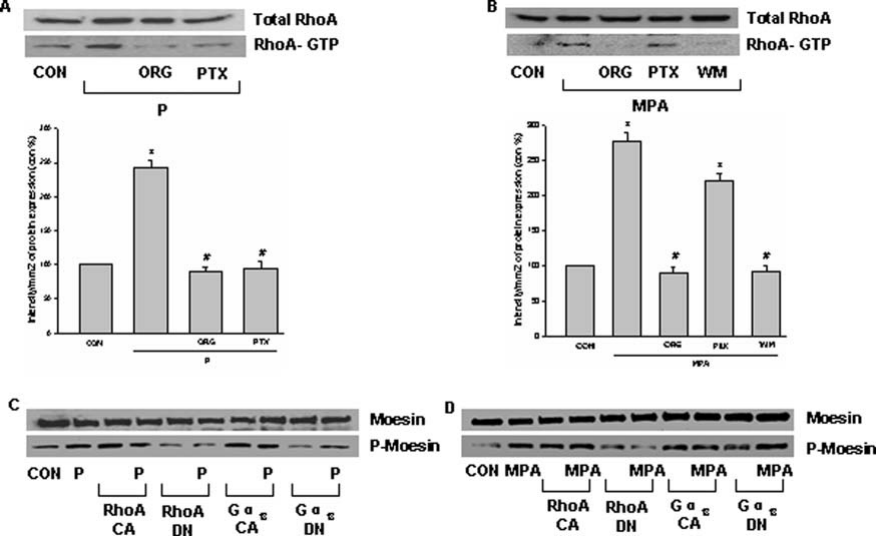
Extra-nuclear signaling of PR to moesin in T47-D cells: RhoA. (A) and (B) RhoA activity was assayed in cells treated with progesterone or MPA (both 100 nM) for 15 min in the presence or absence of the pure PR antagonist ORG 31710 (ORG - 1 µM), of the PI3K inhibitor wortmannin (WM - 30 nM), or of the G protein inhibitor, PTX (100 ng/mL). Active, GTP-bound RhoA was immunoprecipitated with Rhoteckin and subsequently assayed with western analysis with an anti-RhoA Ab (lower boxes). The upper boxes show the total RhoA content in the input. * = P<0.05 vs control, # = P<0.05 vs corresponding progestin. (C) and (D) T47-D cells were either mock-transfected or exposed to constitutively active or dominant-negative RhoA (RhoA CA or RhoA DN) and Gα_13_ (Gα_13_ CA or Gα_13_ DN). Cells were then treated with progesterone or MPA (both 100 nM) for 15 min and wild type and P-moesin were analyzed. The experiments were performed in triplicates and representative images are shown.

The small GTPase RhoA mediates the signaling of a variety of receptors to ERM proteins, including that of sex steroid receptors [12]. Indeed, PR activation in T47-D cells with P or MPA increased the amount of active, GTP-bound RhoA (Fig. 7A–B). In agreement with the previous results, the PR-dependent recruitment of RhoA was mediated by G proteins in the presence of P (Fig. 7A), while it involved PI3K, and not G proteins, in the presence of MPA (Fig. 7B).

Supporting the role of Gα_13_ and RhoA in the signaling of PR, moesin phosphorylation was ligand-independently induced by transient transfection of Gα_13_ (Gα_13_ Q226L) or RhoA (RhoA G14V) constitutively active constructs (Fig. 7C–D). In parallel, transfection of a dominant negative RhoA (RhoA T19N) construct resulted in a significant reduction of P- and MPA-induced moesin phosphorylation (Fig. 7C–D). In line with the previous results, a dominant negative Gα_13_ construct (Gα_13_ Q226L/D294N) decreased the amount of moesin phosphorylation induced by P but not by MPA (Fig. 7C–D).

### Later intracellular events linking activation of PR to moesin: role of the Rho-associated kinase, ROCK-2

**Figure 8:**
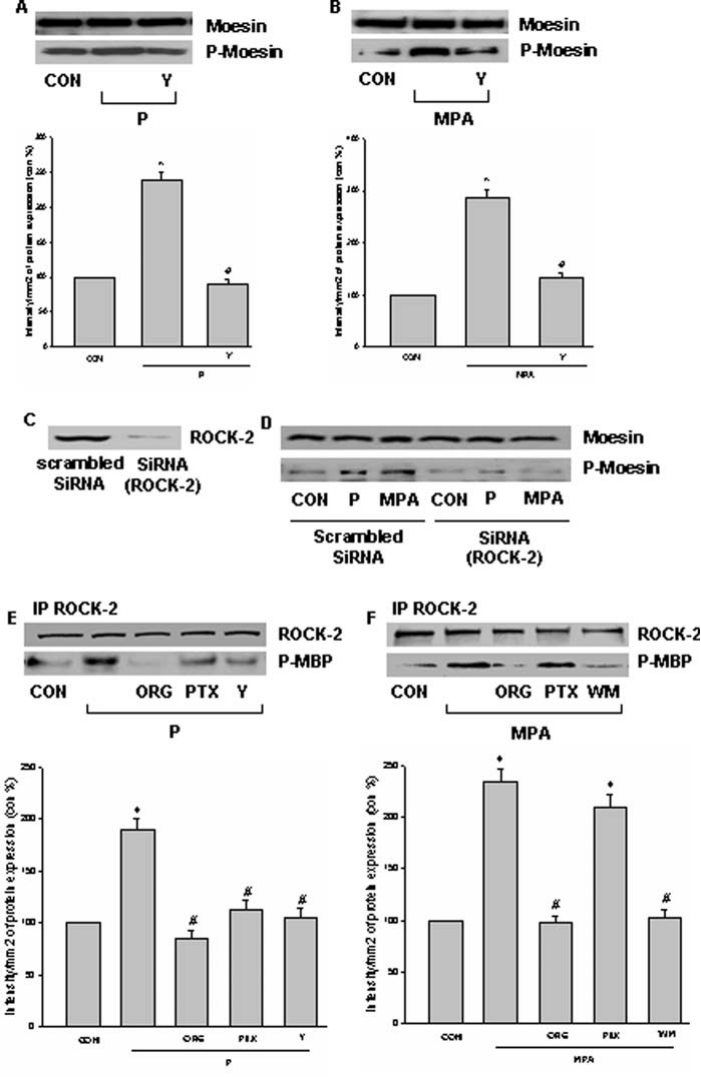
Extra-nuclear signaling of PR to moesin in T47-D cells: ROCK-2. (A) and (B) T47-D cells were exposed for 15 min to progesterone or MPA (both 100 nM) in the presence or absence of the ROCK-2 inhibitor, Y-27632 (Y - 10 µM) and moesin and phosphorylated moesin were assayed with western analysis. * = P<0.05 vs control, # = P<0.05 vs corresponding progestin. (C) T47-D cells were transfected with scrambled siRNA or ROCK-2 target siRNA for 48 h. ROCK-2 protein expression was detected by western blot. (D) Cells were exposed to 100 nM progesterone or MPA for 15 min after transfection with 100 nM target siRNA for ROCK-2 or scrambled siRNA for 48 h. Cell contents of wild-type or phosphorylated moesin are shown. (E) and (F) Cells were treated with progesterone or MPA (both 100 nM) for 15 min in the presence or absence of ORG 31710 (ORG - 1 µM), of wortmannin (WM - 30 nM), of PTX (100 ng/mL) or of Y-27632 (Y - 10 µM). ROCK-2 was immunoprecipitated with a specific Ab and the IPs were used to phosphorylate the bait protein, myelin basic protein (MBP). ROCK-2 kinase activity is shown as the amount of phosphorylated MBP (P-MBP). * = P<0.05 vs control, # = P<0.05 vs corresponding progestin. The experiments were performed in triplicates and representative images are shown.

Blockade of ROCK-2 with the specific inhibitor Y-27632 prevented the PR-dependent moesin activation induced by P or MPA (Fig. 8A–B). In addition, silencing of ROCK-2 with siRNAs (Fig. 8C) prevented the PR-dependent moesin activation induced by both P and MPA (Fig. 8D).

In the presence of progesterone, ROCK-2 was functionally activated, as shown by enhanced Thr-phosphorylation of the bait protein myelin basic protein (MBP) by ROCK-2 immunoprecipitates (IPs) (Fig. 8E). ROCK-2 activation by P was prevented by the PR antagonist ORG 31710 and by the G protein inhibitor, PTX (Fig. 8E). Recruitment of PR by MPA also lead to ROCK-2 activation (Fig. 8F). ORG 31710 and the PI3K inhibitor wortmannin inhibited this action of MPA, while PTX was ineffective (Fig. 8F).

### Intracellular events linking activation of PR to cell migration and invasion

**Figure 9:**
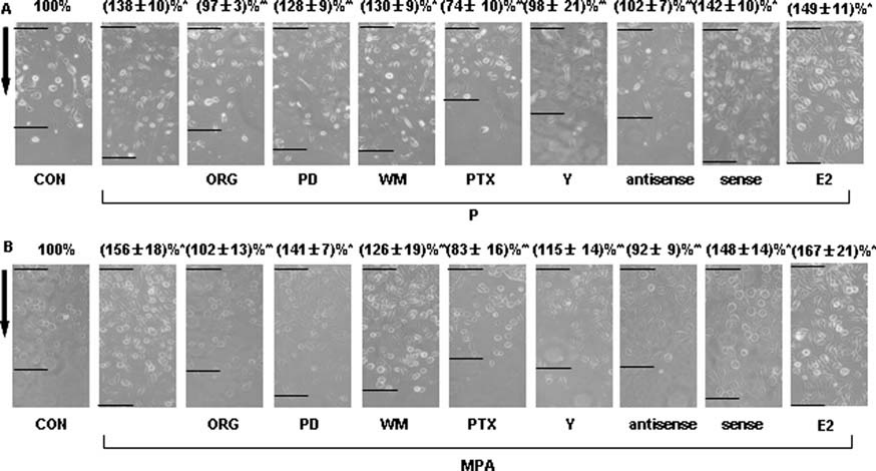
Intracellular signaling mechanisms involved in PR-enhanced T47-D cell migration. (A) and (B) Cells were treated with progesterone or MPA (both 100 nM) for 48 h, in the presence or absence of ORG 31710 (ORG - 1 µM), of PD98059 (PD - 5 µM), of wortmannin (WM - 30 nM), of PTX (100 ng/mL), of Y-27632 (Y - 10 µM) or of 17β - estradiol (E2 - 1 nM). Other cells were transfected with moesin antisense phosphorotioate oligonucleotides (PON) (antisense - 2 µM) or sense PON (sense - 2 µM). Cell migration distances were measured and values are presented as % of control. * = P<0.01 vs. control; ** = P<0.05 vs. progesterone or MPA. The experiments were performed in triplicates and data representing the migration distance of cells from the starting line are expressed as mean±SD. Representative images are shown. The arrows indicate the direction of migration. The upper black lines indicate the starting line and the lower black lines indicate the mean migration distance.

We finally explored the signaling mechanisms implicated in cell migration and invasion. P and MPA-enhanced cell migration was inhibited by blocking PR with the pure PR antagonist ORG 31710 or ROCK with the specific inhibitor Y-27632 (Fig. 9A–B). Inhibition of G proteins resulted in a near-complete blockade of cell migration in the presence of either P or MPA (Fig. 9A–B), consistent with a broader role of G proteins for cell movement, that likely overrides the PR-to-ROCK cascade. Inhibition of PI3K or of MAPK decreased both P- and MPA-promoted cell migration to some extent (Fig. 9A–B). However, a statistically significant reduction of cell migration was found only for the addition of PD98059 to P treatment (Fig. 9A) and for the addition of wortmannin to MPA treatment (Fig. 9B).

**Figure 10:**
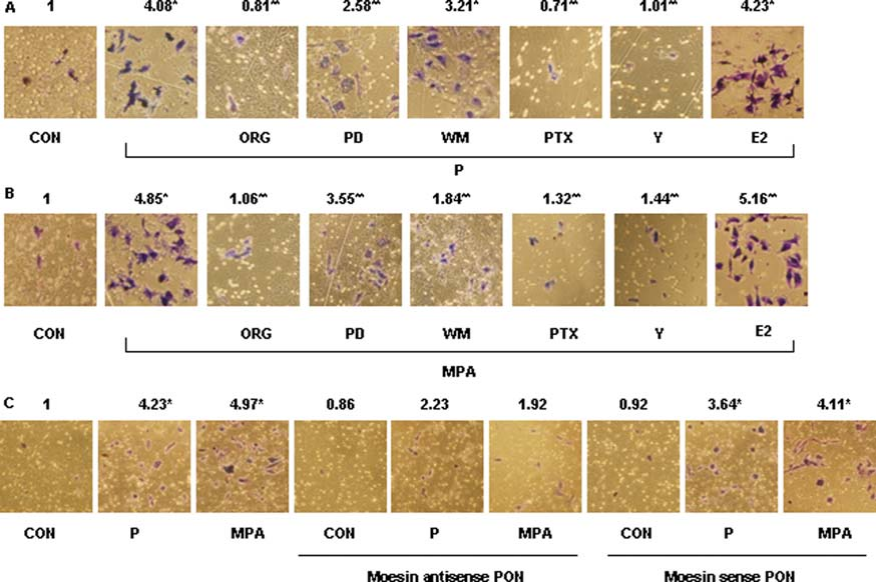
Intracellular signaling steps involved in PR-enhanced T47-D cell invasion. (A) and (B) T47-D cells were treated with progesterone or MPA (both 100 nM) for 24 h, in the presence or absence of the substances as previously indicated or of transfection with moesin antisense phosphorotioate oligonucleotides (PON) (antisense - 2 µM) or sense PON (sense - 2 µM) for 48 h and cell invasion was assayed using invasion chambers. Invading cells were counted in three different central fields of triplicate membranes. Invasion indexes and representative images are shown. * = P<0.01 vs. control, ** = P<0.05 vs P or MPA. The experiments were performed in triplicates. Invasion indexes and representative images are shown.

P or MPA promoted invasion of the matrix by cancer cells (Fig. 10A–B). The invasive behavior induced by P or MPA was prevented by blocking PR, G proteins or ROCK-2 (Fig. 10A–B). Lesser inhibitory effects were found in when the MAPK inhibitor, PD98059 and of the PI3K inhibitor, wortmannin were added to either P or MPA (Fig. 10A–B). Silencing of moesin with antisense oligonucleotides (PON) fully prevented the effects of P and MPA on cell migration and invasion (Fig. 9A–B, 10C), indicating the pivitol role of moesin in these processes.

Estrogen promotes T47-D cell migration and invasion [2]. However, in the presence of 17β-estradiol (E2 - 1 nM), a slight but non-significant additive effects was found during co-treatment with P or MPA (Fig. 9A–B, 10A–B).

## Discussion

Progesterone receptor is a fundamental orchestrator of breast development and function [29], but is also implicated in breast cancer development and progression, although its role in these processes is still to be fully established [1]. Nonetheless, recent evidence from clinical trials [4], [30], [31] suggests that exposure to progesterone may a key factor for breast cancer.

Cancer cells spread locally and metastasize to distant organs and these processes represent the chief cause of morbidity and death [6]. Endocrine therapy using the progesterone receptor (PR) antagonist RU486 prevents the development of mammary tumors and induces the regression of lymph node and lung metastases in mouse breast cancer models [32], [33], supporting a role for PR in these processes. In addition, PR agonists enhance the invasiveness of breast cancer cells by increasing tissue factor or vascular endothelial growth factor expression [34], [35]. However, definitive mechanistic explanations of the effects of PR on breast cancer cell movement or invasion are not available.

Cell movement is a complex and highly integrated process. The formation of a cortical actin complex at specialized membrane structures, such as pseudopodia, lamellipodia and membrane ruffles [36] bridges the cytoskeleton to the extracellular matrix. The actin-binding protein moesin plays a central role in these processes [36], [37].

Our previous findings show indicate that sex steroid receptors, such as the estrogen receptor alpha, control moesin activity in vascular cells [12] as well as in breast cancer cells [2]. In this paper, we discover that PR signals to moesin in breast cancer cells and this leads to rapid actin emodeling that supports horizontal cell movement and invasion of three-dimensional matrices. Estrogen slightly potentiates both progestins-enhanced cell migration and invasion Moesin is required for these tasks, as its silencing results in reduced migration in the presence of progestins. These findings are consistent with previous reports of effects of progesterone on the formation of adhesion structures and on cytoskeletal modifications in other breast cancer cell lines [38].

In the presence of progesterone, PRA interacts with the G protein Gα_13_, therefore recruiting the RhoA/ROCK-2 cascade. This results in moesin phosphorylation and in the morphological changes in the cell. PRB also interacts with Gα_13_, however, this interaction is not dependent on the presence of a ligand, nor it recruits the Gα_13_/RhoA/ROCK-2 cascade.

The finding of the interaction of PRA and Gα_13_ is consistent with previous reports of PR signaling through Gα_i_ and Gβγ [25], [39], [40]. Gα_13_ belongs to the G_12_ family that is critical for cell movement and plays an important role in metastasis [24], [41]. Indeed, expression of activated Gα_13_ in breast cancer cells increases cell invasion [42]. Our finding of the recruitment of Gα_13_ by PRA thus provides a mechanistic explanation for the progesterone-dependent breast cancer cell migration and invasion.

Recent work indicates the existence of membrane-localized progesterone receptors [25], [26]. As Gα_13_ is a cell membrane protein, the finding that moesin phosphorylation can be induced by a membrane-impermeable form of progesterone might be compatible with the recruitment of PRA at this level. However, this is just suggestive and not conclusive. The identification of the cellular site of PR/Gα_13_ interaction is therefore not solved and will be the aim of future studies.

In normal mammary epithelial cells PR isoforms are co-expressed equivalently. However, PR isoform predominance, especially PRA predominance or an increased PRA/PRB ratio, is found in a high proportion of breast cancers and correlates to invasive behaviour [43], [44]. Moreover, an increased PRA/PRB ratio in breast cancer cells has been shown to induce changes in cell morphology and the loss of cell adhesion in response to progesterone receptor agonists, along with a membrane-to-cytoplasm redistribution of the ERM protein, ezrin [45]. Increasing PRA levels in breast cancer cells is also associated with altered expression of genes associated with regulation of cell shape and adhesion [46]. More recently PRB (but not PRA) has been shown to localize to the cytoplasm in response to progesterone and thus to interact with c-Src. This leads to the activation of MAPK and to subsequent up-regulation of cyclin D1 in breast cancer cells [19]. To this extent, our finding of a divergent ability of PRA and PRB to recruit the RhoA/ROCK-2 cascade stands in favour of a different role of the two receptor isoforms during cell migration and invasion.

PR-dependent recruitment of the MAPK and PI3K/Akt pathways in the presence of natural progesterone does not seem to be involved in moesin activation, but the inhibition of these two pathways decreases cell migration and invasion in T47-D cells exposed to progesterone. This implies that moesin is not the exclusive tool mediating cell migration and invasion in the presence of this PR agonist, which is not surprising. On the other hand, PRA does not interact with Gα_13_ when bound by the synthetic progestin, MPA, indicating a high degree of specificity of this signaling event. Indeed, when MPA hits PRA or PRB, the Src/PI3K/Akt pathway is rapidly recruited. This ultimately leads to the activation of RhoA and ROCK-2 and, finally, of moesin. It is possible that when engaged by MPA, PRs may be driven to form a functional signaling module with Src and PI3K, where activated PI3K would lead to recruitment of RhoA, as shown in other cells [47]. In analogy, ER, Src and PI3K are reportedly organized into a similar complex to mediate rapid signaling of estrogens in endothelial cells [48].

While PI3K is critical for moesin activation by MPA, G proteins and MAPK are still relevant for MPA-induced cell migration. A similar result is found for breast cancer invasion of three-dimensional matrices, where the inhibition of G proteins, ROCK and MAPK all result in a significant decrease of progesterone-induced cell invasion, notwithstanding the fact that MAPK are not required for moesin activation by progesterone. These apparent discrepancies are likely due to the complexity of the processes of cell movement and invasion, that are controlled by multiple internal and external signals [49].

Some of the present results point out that PR signals differently when engaged by different agonists, such as P or MPA, and this maybe responsible for their discrepant actions on some specific endpoints, such as their different impacts on breast cancer subtype [16]. The basis for this phenomenon is not currently understood. Progestins act differentially in part due to the ability to engage other steroid receptors [50]. MPA is able to bind the glucocorticoid receptor (GR) and this explains some effects of this progestin in endothelial cells [21]. However, GR is not responsible for the MPA-dependent activation of moesin, possibly indicating that conformational differences in PR might explain the differential recruitment of signaling pathways in the presence of the two ligands.

In conclusion, we show that PR is implicated in breast cancer cell migration and invasion. Recruitment of PR by P or MPA leads to rapid extra-nuclear signaling to actin, associated to the rearrangement of the cytoskeleton and the formation of pseudopodia and membrane ruffles. These changes increase breast cancer cell invasion of the surrounding environment. PR signaling seems to be ligand-specific, as in the presence of progesterone PR signals to RhoA and ROCK-2 through the activation of Gα_13_, while in the presence of MPA PR uses c-Src and PI3K. These observations help to understand the role of progesterone receptor signaling in breast cancer spread and could provide new molecular targets for breast cancer treatment.

## Materials and Methods

### Cell cultures and treatments

T47-D breast cancer cells were incubated in phenol red-free RPMI 1640 medium containing 10% fetal calf serum (FCS), 0.2 UI/mL insulin, L-glutamine and penicillin streptomycin under a 5% CO_2_ atmosphere at 37°C. Before experiments investigating non-transcriptional effects, cells were kept in phenol red-free DMEM containing no FBS for 8 hours. Whenever an inhibitor was used, the compound was added 30 minutes before starting the treatments. Progesterone, medroxyprogesterone acetate, 17β-estradiol, hydrocortisone, pertussis toxin, Y-27632, PD98059, wortmannin, actinomycin D and cycloheximide were from Sigma-Aldrich (Saint-Louis, MO). 4-pregnen-3, 20-dione3-O-carboxymethyloxime: BSA (P-BSA) was from Steraloids (Steraloids incorporation, Newport, RI). 4-amino-5-(4-chlorophenyl)-7-(t-butyl) pyrazolo (3,4-d) pyrimidine (PP2) was from Calbiochem (EMD Biosciences, Germany). ORG 31710 was a kind gift of Dr. Lenus Kloosterboer, from Organon Akzo Nobel (Oss, The Netherlands).

### Immunoblottings

Cell lysates were separated by SDS-PAGE. Antibodies used were: moesin (clone 38, Transduction Laboratories, Lexington, KY), Thr^558^-P-moesin (sc-12895, Santa Cruz Biotechnology, Santa Cruz, CA), PR (sc-539, Santa Cruz), Tyr^204^-P-ERK (sc-7969, Santa Cruz), Gα_13_ protein (sc-410, Santa Cruz), ERK1/ERK2 (444944, Calbiochem-Novabiochem Corporation, San diego, CA), Thr^34^-P-Akt (07-789, Upstate, Lake Placid, NY), Akt (9272, Cell signalling technology, Danvers, MA). Primary and secondary Abs were incubated with the membranes with standard technique [51]. Immunodetection was accomplished using enhanced chemiluminescence. Chemiluminescence was acquired with a quantitative digital imaging system (Quantity One, BioRad, Hercules, CA) allowing to check for saturation. Overall emitted photons were quantified for each band, particularly for loading controls, which were homogeneously loaded.

### Kinase assays

T47-D cells were harvested in 20 mM Tris-HCl, 10 mM EDTA, 100 mM NaCl, 0.5% IGEPAL and 0.1 mg/mL PMSF. Equal amounts of cell lysates were immunoprecipitated with Rhotekin RBD agarose (14-383, upstate) *vs*. GTP-RhoA or an Ab *vs*. ROCK-2 (C-20, Santa Cruz). The IPs were washed three times with buffer containing 20 mM Tris-HCl, 10 mM EDTA, 150 mM NaCl, 0.1% IGEPAL and 0.1 mg/mL PMSF. For ROCK-2 activity assay, two additional washes were performed in kinase assay buffer (20 mM MOPS, 25 mM β-glycerophosphate, 5 mM EGTA, 1 mM DTT) and the samples were therefore resuspended in this buffer. 5 µg of de-phosphorylated myelin basic protein (Upstate) together with 500 µM ATP and 75 mM MgCl_2_ were added to each sample and the reaction was started at 30°C for 20 min. The reaction was stopped on ice and by resuspending the samples in Laemmli Buffer. The samples were separated with SDS-PAGE and Western analysis was performed using antibodies recognizing RhoA (sc-418, Santa Cruz) or Thr^98^-P-myelin basic protein (05-429, Upstate).

### Cell immunofluorescence

T47-D breast cancer cells were grown on coverslips and exposed to treatments. Cells were fixed with 4% paraformaldehyde for 30 min and permeabilized with 0.1% Triton X for 5 min. Blocking was performed with 3% normal serum for 20 min. Cells were incubated with antibodies against Gα_13_ or PR (sc-418, Santa Cruz). After washing the nuclei were counterstained with 4′-6-diamidino-2-phenylindole (DAPI) (Sigma) and actin was stained with Texas Red-phalloidin (Sigma). The coverslips were mounted with Vectashield mounting medium (Vector Laboratories, Burlingame, CA). Immunofluorescence was visualized using an Olympus BX41 microscope and recorded with a high-resolution DP70 Olympus digital camera. Pictures were photographed. Cell membrane thickness and the gray level of extracellular area, cell membrane as well as cytoplasm were quantitated using Leica QWin image analysis and image processing software (Leica Microsystems, Wetzlar, Germany).

### Transfection experiments

On-TARGETplus SMARTpool siRNA reagents against human MAPK (NM-138957), ROCK-2 (NM-004850), Src (NM-198291) and control siRNA (D-001810-01-05) were purchased from Dharmacon (Thermo Fisher Scientific Inc, USA). T47-D cells were transfected with siRNA using Lipofectamine (Invitrogen) according to the protocol. Cells (40% confluent) were serum-starved for 1 h followed by incubation with 100 nM target siRNA or control siRNA for 6 h in serum-free media. The serum-containing media was then added (10% serum final concentration) for 42 h before experiments and/or functional assays were conducted. Target protein silencing was assessed through protein analysis up to 48 h after transfection.

Each plasmid (15 µg) was transfected into T47-D breast cancer cells using the Lipofectamine (Invitrogen) according to the manufacturer's instructions. The transfected plasmids were as follows: Gα_13_ Q226L, Gα_13_ Q226L/D294N, RhoA T19 and RhoA G14V, p85α or dominant-negative p85α (Δp85α). These constructs were obtained from the Guthrie cDNA Resource Center (www.cdna.org). Plasmids for CMV human progesterone receptor A (hPR-A, # 95) and B (hPR-A, # 90) were provided by Dean P. Edwards (Baylor college of medicine, USA). All the inserts were cloned in pcDNA3.1+. As control, parallel cells were transfected with empty pcDNA3.1+ plasmid. Cells (60–70% confluent) were treated 24 h after transfection, and cellular extracts were prepared according to the experiments to be performed.

Validated antisense phosphorotioate oligonucleotides (S-modified) (PONs) complementary to the 1–15 position of the human moesin gene coding region were obtained from Dharmacon. The sequence was 5′-TACGGGTTTTGCTAG-3′ for moesin antisense PON. The complementary sense PON was used as control (5′-CTAGCAAAACCCGTA-3′). Transfections were performed on subconfluent T47-D cells. PONs were resuspended in serum-free medium with Lipofectamine (Invitrogen) and added to the culture medium every 12 h at the final concentration of 4 µM. Every 24 h, cells were washed and fresh medium supplemented with 4 µM PONs was added. Moesin silencing was assessed through protein analysis up to 48 h after transfection.

### G-actin /F-actin in vivo assay

G-actin/F-actin in vivo assay kit was purchased from Cytoskeleton Inc (# BK037, Denver, USA). This kit is used to determine accurately the amount of filamentous actin (F-actin) content versus free globular-actin (G-actin) content in a cell population. In brief, confluent T47-D cells were harvested with 37°C warm lysis and F-actin stabilization buffer (50 mM PIPES, 50 mM KCl, 5 mM MgCl_2_, 5 mM EGTA, 5% glycerol, 0.1% Nonidet P40, 0.1% Triton X-100, 0.1% Tween 20, 0.1% 2-mercapto-ethanol, 0.001% antifoam C, 1 mM ATP) after required treatments. Total protein concentration was determined by standard method. Positive and negative controls were set by adding F-actin enhancing solution (phalloidin, 1 µM) or F-actin depolymerization solution (10 µM cytochalasin-D) to the lysates, respectively. The lysates were incubated at 37°C for 10 min, followed by a centrifuge at 2000 rpm for 5 min to pellet and discard unbroken cells. Supernatant were centrifuged at 10,000×*g* for 1 h at 37°C. After that, supernatant and pellet were both collected. Pellets were resuspended to the same volume as the supernatant using ice cold distilled water plus F-actin depolymerization solution (10 µM cytochalasin-D) and put on ice for 1 h to dissociate F-actin. According to the protein concentration previously measured, equivalent volumes of supernatant and dissolved pellet were loaded to run Western blot and G-actin/F-actin ratio was quantitiated using the quantitative digital imaging system.

### Cell migration assays

Cell migration was assayed with razor scrape assays as previously described [12]. Briefly, a razor blade was pressed through the confluent T47-D breast cancer cell monolayer into the plastic plate to mark the starting line. T47-D cells were swept away on one side of that line. Cells were washed, and 2.0 mL of DMEM containing steroid-deprived FBS and gelatin (1 mg/mL) were added. Cytosine β-D-arabinofuranoside hydrochloride (Sigma) (10 µM), a selective inhibitor of DNA synthesis which doesn't inhibit RNA synthesis was used 1 h before the test substance was added. Migration was monitored for 48 hours. Every 12 h fresh medium and treatment were replaced. Cells were digitally imaged and migration distance was measured by using phase-contrast microscopy.

### Cell invasion assays

Cell invasion were assayed following the standard method by using the BD BioCoatTM Growth Factor Reduced (GFR) Matrigel^TM^ Invasion Chamber (BD Bioscience, USA). In brief, after rehydrating the GFR Matrigel inserts, the test substance was added to the wells. An equal number of Control Inserts (no GFR Matrigel coating) were prepared as control. 0.5 mL of T47-D cell suspension (2.5×10^4^ cells/mL) were added to the inside of the inserts. The chambers were incubated for 24 h at 37°C, 5% CO_2_ atmosphere. After incubation, the non-invading cells were removed from the upper surface of the membrane using cotton tipped swabs. Then the cells on the lower surface of the membrane were stained with Diff-Quick stain. The invading cells were observed and photographed under the microscope at 100× magnification. Cells were counted in the central field of triplicate membranes. The invasion index was calculated as the % invasion test cell/ % invasion control cell.

### Statistical analysis

All values are expressed as mean±SD. Statistical differences between mean values were determined by ANOVA, followed by the Fisher's protected least significance difference (PLSD). All differences were considered significant at P<0.05.

## References

[1] Yager JD, Davidson NE (2006). Estrogen carcinogenesis in breast cancer.. N Engl J Med.

[2] Giretti MS, Fu XD, De Rosa G, Sarotto I, Baldacci C (2008). Extra-nuclear signalling of estrogen receptor to breast cancer cytoskeletal remodelling, migration and invasion.. PLoS ONE.

[3] Lee S, Kolonel L, Wilkens L, Wan P, Henderson B (2006). Postmenopausal hormone therapy and breast cancer risk: the Multiethnic Cohort.. Int J Cancer.

[4] Rossouw JE, Anderson GL, Prentice RL, LaCroix AZ, Kooperberg C (2002). Risks and benefits of estrogen plus progestin in healthy postmenopausal women: principal results From the Women's Health Initiative randomized controlled trial.. Jama.

[5] Anderson GL, Limacher M, Assaf AR, Bassford T, Beresford SA (2004). Effects of conjugated equine estrogen in postmenopausal women with hysterectomy: the Women's Health Initiative randomized controlled trial.. Jama.

[6] Weigelt B, Peterse JL, van't Veer LJ (2005). Breast cancer metastasis: markers and models.. Nat Rev Cancer.

[7] Carragher NO, Frame MC (2004). Focal adhesion and actin dynamics: a place where kinases and proteases meet to promote invasion.. Trends Cell Biol.

[8] Pawlak G, Helfman DM (2001). Cytoskeletal changes in cell transformation and tumorigenesis.. Curr Opin Genet Dev.

[9] Louvet-Vallee S (2000). ERM proteins: from cellular architecture to cell signaling.. Biol Cell.

[10] Tsukita S, Yonemura S (1999). Cortical actin organization: lessons from ERM (ezrin/radixin/moesin) proteins.. J Biol Chem.

[11] Oshiro N, Fukata Y, Kaibuchi K (1998). Phosphorylation of moesin by rho-associated kinase (Rho-kinase) plays a crucial role in the formation of microvilli-like structures.. J Biol Chem.

[12] Simoncini T, Scorticati C, Mannella P, Fadiel A, Giretti MS (2006). Estrogen receptor alpha interacts with Galpha13 to drive actin remodeling and endothelial cell migration via the RhoA/Rho kinase/moesin pathway.. Mol Endocrinol.

[13] Schindler AE, Campagnoli C, Druckmann R, Huber J, Pasqualini JR (2003). Classification and pharmacology of progestins.. Maturitas.

[14] Stanczyk FZ (2003). All progestins are not created equal.. Steroids.

[15] Fournier A, Berrino F, Riboli E, Avenel V, Clavel-Chapelon F (2005). Breast cancer risk in relation to different types of hormone replacement therapy in the E3N-EPIC cohort.. Int J Cancer.

[16] Fournier A, Fabre A, Mesrine S, Boutron-Ruault MC, Berrino F (2008). Use of different postmenopausal hormone therapies and risk of histology- and hormone receptor-defined invasive breast cancer.. J Clin Oncol.

[17] Kastner P, Krust A, Turcotte B, Stropp U, Tora L (1990). Two distinct estrogen-regulated promoters generate transcripts encoding the two functionally different human progesterone receptor forms A and B.. Embo J.

[18] Saitoh M, Ohmichi M, Takahashi K, Kawagoe J, Ohta T (2005). Medroxyprogesterone acetate induces cell proliferation through up-regulation of cyclin D1 expression via phosphatidylinositol 3-kinase/Akt/nuclear factor-kappaB cascade in human breast cancer cells.. Endocrinology.

[19] Boonyaratanakornkit V, McGowan E, Sherman L, Mancini MA, Cheskis BJ (2007). The role of extranuclear signaling actions of progesterone receptor in mediating progesterone regulation of gene expression and the cell cycle.. Mol Endocrinol.

[20] Fu XD, Simoncini T (2007). Non-genomic sex steroid actions in the vascular system.. Semin Reprod Med.

[21] Simoncini T, Mannella P, Fornari L, Caruso A, Willis MY (2004). Differential signal transduction of progesterone and medroxyprogesterone acetate in human endothelial cells.. Endocrinology.

[22] Thomas CP, Liu KZ, Vats HS (2006). Medroxyprogesterone acetate binds the glucocorticoid receptor to stimulate alpha-ENaC and sgk1 expression in renal collecting duct epithelia.. Am J Physiol Renal Physiol.

[23] Faivre EJ, Lange CA (2007). Progesterone receptors upregulate Wnt-1 to induce epidermal growth factor receptor transactivation and c-Src-dependent sustained activation of Erk1/2 mitogen-activated protein kinase in breast cancer cells.. Mol Cell Biol.

[24] Offermanns S, Mancino V, Revel JP, Simon MI (1997). Vascular system defects and impaired cell chemokinesis as a result of Galpha13 deficiency.. Science.

[25] Karteris E, Zervou S, Pang Y, Dong J, Hillhouse EW (2006). Progesterone signaling in human myometrium through two novel membrane G protein-coupled receptors: potential role in functional progesterone withdrawal at term.. Mol Endocrinol.

[26] Zhu Y, Bond J, Thomas P (2003). Identification, classification, and partial characterization of genes in humans and other vertebrates homologous to a fish membrane progestin receptor.. Proc Natl Acad Sci U S A.

[27] Boonyaratanakornkit V, Scott MP, Ribon V, Sherman L, Anderson SM (2001). Progesterone receptor contains a proline-rich motif that directly interacts with SH3 domains and activates c-Src family tyrosine kinases.. Mol Cell.

[28] Koga F, Xu W, Karpova TS, McNally JG, Baron R (2006). Hsp90 inhibition transiently activates Src kinase and promotes Src-dependent Akt and Erk activation.. Proc Natl Acad Sci U S A.

[29] Humphreys RC, Lydon JP, O'Malley BW, Rosen JM (1997). Use of PRKO mice to study the role of progesterone in mammary gland development.. J Mammary Gland Biol Neoplasia.

[30] Ross RK, Paganini-Hill A, Wan PC, Pike MC (2000). Effect of hormone replacement therapy on breast cancer risk: estrogen versus estrogen plus progestin.. J Natl Cancer Inst.

[31] Schairer C, Lubin J, Troisi R, Sturgeon S, Brinton L (2000). Menopausal estrogen and estrogen-progestin replacement therapy and breast cancer risk.. Jama.

[32] Poole AJ, Li Y, Kim Y, Lin SC, Lee WH (2006). Prevention of Brca1-mediated mammary tumorigenesis in mice by a progesterone antagonist.. Science.

[33] Vanzulli SI, Soldati R, Meiss R, Colombo L, Molinolo AA (2005). Estrogen or antiprogestin treatment induces complete regression of pulmonary and axillary metastases in an experimental model of breast cancer progression.. Carcinogenesis.

[34] Kato S, Pinto M, Carvajal A, Espinoza N, Monso C (2005). Progesterone increases tissue factor gene expression, procoagulant activity, and invasion in the breast cancer cell line ZR-75-1.. J Clin Endocrinol Metab.

[35] Hyder SM, Chiappetta C, Stancel GM (2001). Pharmacological and endogenous progestins induce vascular endothelial growth factor expression in human breast cancer cells.. Int J Cancer.

[36] Pollard TD, Borisy GG (2003). Cellular motility driven by assembly and disassembly of actin filaments.. Cell.

[37] Hughes SC, Fehon RG (2007). Understanding ERM proteins–the awesome power of genetics finally brought to bear.. Curr Opin Cell Biol.

[38] Lin VC, Ng EH, Aw SE, Tan MG, Ng EH (2000). Progesterone induces focal adhesion in breast cancer cells MDA-MB-231 transfected with progesterone receptor complementary DNA.. Mol Endocrinol.

[39] Machelon V, Nome F, Grosse B, Lieberherr M (1996). Progesterone triggers rapid transmembrane calcium influx and/or calcium mobilization from endoplasmic reticulum, via a pertussis-insensitive G-protein in granulosa cells in relation to luteinization process.. J Cell Biochem.

[40] Lutz LB, Kim B, Jahani D, Hammes SR (2000). G protein beta gamma subunits inhibit nongenomic progesterone-induced signaling and maturation in Xenopus laevis oocytes. Evidence for a release of inhibition mechanism for cell cycle progression.. J Biol Chem.

[41] Kelly P, Stemmle LN, Madden JF, Fields TA, Daaka Y (2006). A role for the G12 family of heterotrimeric G proteins in prostate cancer invasion.. J Biol Chem.

[42] Kelly P, Moeller BJ, Juneja J, Booden MA, Der CJ (2006). The G12 family of heterotrimeric G proteins promotes breast cancer invasion and metastasis.. Proc Natl Acad Sci U S A.

[43] Mote PA, Bartow S, Tran N, Clarke CL (2002). Loss of co-ordinate expression of progesterone receptors A and B is an early event in breast carcinogenesis.. Breast Cancer Res Treat.

[44] Hopp TA, Weiss HL, Hilsenbeck SG, Cui Y, Allred DC (2004). Breast cancer patients with progesterone receptor PR-A-rich tumors have poorer disease-free survival rates.. Clin Cancer Res.

[45] McGowan EM, Weinberger RP, Graham JD, Hill HD, Hughes JA (2003). Cytoskeletal responsiveness to progestins is dependent on progesterone receptor A levels.. J Mol Endocrinol.

[46] Graham JD, Yager ML, Hill HD, Byth K, O'Neill GM (2005). Altered progesterone receptor isoform expression remodels progestin responsiveness of breast cancer cells.. Mol Endocrinol.

[47] Saci A, Carpenter CL (2005). RhoA GTPase regulates B cell receptor signaling.. Mol Cell.

[48] Haynes MP, Li L, Sinha D, Russell KS, Hisamoto K (2003). Src kinase mediates phosphatidylinositol 3-kinase/Akt-dependent rapid endothelial nitric-oxide synthase activation by estrogen.. J Biol Chem.

[49] Kamps AR, Coffman CR (2005). G protein-coupled receptor roles in cell migration and cell death decisions.. Ann N Y Acad Sci.

[50] Sitruk-Ware R (2003). Progestins in hormonal replacement therapy (HRT): new molecules, risks and benefits.. Ann Endocrinol (Paris).

[51] Simoncini T, Hafezi-Moghadam A, Brazil DP, Ley K, Chin WW (2000). Interaction of oestrogen receptor with the regulatory subunit of phosphatidylinositol-3-OH kinase.. Nature.

